# The success rate and complications of midline catheters in pediatric outpatient parenteral antibiotic therapy (OPAT)

**DOI:** 10.1007/s00431-024-05432-7

**Published:** 2024-01-16

**Authors:** Urban Fläring, Henrik Lundevall, Åke Norberg, Andreas Andersson

**Affiliations:** 1 Department of Pediatric Perioperative Medicine and Intensive Care, Astrid Lindgren Children’s Hospital, Karolinska University Hospital, Stockholm, Sweden; 2 Department of Physiology and Pharmacology, Karolinska Institutet, Stockholm, Sweden; 3 Department of Clinical Science, Intervention and Technology, Karolinska Institutet, Stockholm, Sweden

**Keywords:** Adverse events, Central venous catheter, Midline catheter, Outpatient parenteral antimicrobial therapy, Pediatric, Vascular access

## Abstract

The use of outpatient parenteral antimicrobial therapy (OPAT) for children has several advantages, including reduced length of hospital stay and costs. A reliable vascular access is key to delivering safe and effective pediatric OPAT. In recent years, midline catheters (MC) have been increasingly used for short-term intravenous antibiotic therapy in children. However, there are no studies investigating the use of MCs in the OPAT setting. The main aim of this paper was to evaluate the success and complications of using MCs for pediatric OPAT. This was a retrospective cohort study from a tertiary academic pediatric hospital. All MCs inserted at the hospital and used for OPAT were eligible for study inclusion. The primary objective was to describe the percentage of patients able to complete OPAT without the need for additional venous access. Forty-one MCs were included in the study. Patient mean (SD) age was 5.9 (4.9) years. In 31 cases (76%, 95% CI 62–86%), the iv therapy could be successfully completed using only the MC. Imbalances between the groups suggested unfavorable outcome for saphenous vein catheters as well as for shorter and smaller-sized catheters. Fourteen patients (34%) were subjected to a MC-related complication. Pain on injection in the MC was the most frequent complication (*n* = 10, 24%).

*   Conclusion*: Midline catheters could be an alternative to central venous access for pediatric OPAT. Avoiding saphenous vein insertion and using longer and larger-sized catheters could increase MC success rate. No severe MC-related complication was found. Further randomized studies comparing different catheter types are needed.

**Table:** 

**What is Known:** *• For selected patients, pediatric outpatient parenteral antimicrobial therapy (OPAT) is safe and provides health-economic, psychosocial, and medical advantages compared to in-hospital care.* *• A reliable venous access is one of the key factors to the success of OPAT, but this can be a challenge in children.*	
**What is New:** *• Using midline catheters, 76% of patients could complete their intended iv therapy without the need for additional venous access. Avoiding saphenous vein insertion and using longer and larger-sized catheters could increase the success rate.* *• Thirty-four percent of catheters were subject to some kind of complication, the most common being pain on injection in the catheter.*

**What is Known:**

*• For selected patients, pediatric outpatient parenteral antimicrobial therapy (OPAT) is safe and provides health-economic, psychosocial, and medical advantages compared to in-hospital care.*

*• A reliable venous access is one of the key factors to the success of OPAT, but this can be a challenge in children.*

**What is New:**

*• Using midline catheters, 76% of patients could complete their intended iv therapy without the need for additional venous access. Avoiding saphenous vein insertion and using longer and larger-sized catheters could increase the success rate.*

*• Thirty-four percent of catheters were subject to some kind of complication, the most common being pain on injection in the catheter.*

## Introduction

The use of outpatient parenteral antimicrobial therapy (OPAT) has several advantages for pediatric patients as well as for the health care system. Family life and routines are better maintained, thereby reducing the negative psychosocial impact of hospitalization [1]. The length of hospital stay can be reduced, and OPAT has been shown to improve patient flow and to be cost-effective [2, 3]. Moreover, the risk of nosocomial infections can be reduced [4].

Even though OPAT has been shown to be safe and effective, there are specific considerations to be made for children receiving OPAT. Complications related to the vascular access are common in children, and failure of the venous access can lead to interruptions in vital treatments as well as the need for hospital readmission [5]. Line-related problems are also one of the aspects of care that parents to children receiving OPAT worry about [6].

Proper selection of intravenous access is crucial to the success of pediatric OPAT. Peripheral intravenous catheters (PIVC) have a shorter lifespan than for adults and can often not be used to complete iv therapy lasting longer than a few days. Central venous catheters (CVC) and PICC lines are often used for pediatric OPAT, but insertion of these catheter types is resource-demanding, often requiring general anesthesia and x-ray confirmation of tip position. Moreover, there are risks of complications such as central vein thrombosis, bleeding, and pneumothorax [7–10]. Midline catheters (MC) are longer PIVCs where the catheter tip does not reach the central circulation [11]. The dwell time is longer than for traditional PIVCs, the rate of catheter-related infections seems to be low [12], and MC insertion can often be achieved using only light sedation.

MCs have several potential benefits compared to central venous access, and the use of MCs for short-term peripheral iv therapy has increased in pediatric care in recent years. Possibly, MCs could be a safe and less resource-demanding option to central venous access for pediatric OPAT. However, the data describing the safety and expected dwell time for pediatric MCs is scarce, and current pediatric vascular access guidelines have highlighted the need for scientific data on this topic [13, 14]. A prospective study from our group recently showed that when using MCs, 78% of children admitted to the hospital did not need additional venous access to complete short-term iv therapy [12]. However, it is unclear if this data is valid for children receiving home care. Children receiving home care are more active, thereby potentially increasing the risk of extravasation or dislodging of the catheter. Catheter hygiene standards can be more difficult to uphold outside the hospital, and catheters in home care are less frequently inspected and flushed by health care personnel compared to catheters used in hospital. Recent British and American guidelines on OPAT have highlighted the need for more data regarding the use of MCs in pediatric OPAT [15, 16].

To better understand the role for pediatric midline catheters in pediatric OPAT, we conducted a retrospective study investigating the efficacy, dwell time, and complications of MCs used for OPAT at our institution.

## Methods

The study was approved by the Regional Ethics Review Board in Stockholm (reference no. 2023–01643-01). The Ethics Review Board waived the necessity of obtaining an informed consent from parents or children.

This retrospective observational cohort study was conducted at the Department of Paediatric Perioperative Medicine and Intensive Care at Karolinska University Hospital, Stockholm, Sweden. All patients (0–18 years) that received a MC from January 1, 2019, to March 31, 2023, were eligible for inclusion if the MC was used for OPAT through the Hospital in the Home service. Patients were administered antibiotics in the home environment by dedicated nurses. All catheters were evaluated individually. Only the initial catheter was included for every OPAT episode, meaning that if the first MC failed, subsequent MCs were not included in the study. According to institutional guidelines, midline insertion was routinely performed under sterile conditions using the Seldinger technique and with real-time ultrasound guidance. At our institution, MCs are inserted by all pediatric anesthetists, and not by a vascular access team. MC is one of the vascular access options for peripheral iv therapy lasting 5–14 days. The choice of catheter type was made by the inserting anesthetist after discussion with the referring physician.

The choice of catheter was at the clinician’s discretion, but the institutional recommendation was to choose a catheter size that keeps the catheter/vein diameter ratio < 0.33. The catheters used in this study were either Vygon Leaderflex (Vygon, Ecouen, France) catheters (2 Fr or 3 Fr, length 4–8 cm) or Vygon Seldipur Smartmidline catheters (3Fr or 4Fr, length 6–12 cm). The length of the MC was determined by the clinician inserting the catheter. For arm vein catheters, tip position in the axillary vein is recommended. For saphenous vein catheters, the recommendation is to use the longest MC available in the selected size (2 Fr MC = 6 cm, 3 Fr MC = 8 cm, 4 Fr = 12 cm). MCs were either sutured or secured with Statlock (Becton Dickinson; Franklin Lakes, NJ, USA), a suture-less securement device. A sterile Tegaderm™ (3 M Healthcare, St. Paul, MN, USA) was applied over the catheter and the insertion point. According to institutional guidelines, sterile dressings were changed every 7 days. After antibiotic administration, catheters were flushed with normal saline; the institutional recommendation is 1 ml/kg of saline. Continuous infusions or locking solutions were not used. The decision to remove a dysfunctional catheter was made by the Hospital in the Home physician.

Study data were retrieved from our electronic patient data management system (Take Care; CompuGroup, Stockholm, Sweden) using standardized collection forms. Catheter-related data including catheter size, length, vein insertion site and side, dwell time, and complications were collected. Patient age, body weight, gender, medical diagnosis, and length of hospital stay were also recorded.

The primary objective of this study was to describe the success rate of MCs defined as completion of iv therapy without the need for additional venous access. MC failure was defined as the need for an additional venous catheter to complete the intended therapy. Furthermore, we aimed to investigate catheter dwell time, the overall rate of catheter complications, and the need for hospital readmission due to catheter complications.

### Statistical analysis

D’Agostino and Pearson omnibus normality test was used to evaluate normality. Normally distributed data are presented as mean (standard deviation (SD)), and non-parametric data are presented as median and interquartile range (IQR). Categorical and ordinal variables are presented as *n* (%), and the 95% confidence intervals (CI) were calculated for the primary objectives of the study according to the Wilson/Brown method. For the groups with MC failure or MC success, absolute standardized differences (ASDs) were calculated for baseline data using the stddiff package for R statistical software. An ASD larger than 1.96 × sqrt((*n*1 + *n*2)/(*n*1 x *n*2)), where *n*1 and *n*2 are the two group samples, was interpreted as an imbalance between the groups [17]. This method was also applied to catheter data. Analyses were performed using GraphPad Prism 8 (GraphPad Software, Inc.7825 Fay Avenue, Suite 230 La Jolla, CA 92037, USA) and R version 4.2.1 (The R Foundation for Statistical Computing).

## Results

Forty-one MCs used for OPAT in 39 patients were included in the study. Four MCs were used for OPAT but were inserted after failure of an initial MC and hence were not included in the study.

### Cohort characteristics

Patient characteristics are given in Table 1. Patient mean (SD) age was 5.9 (4.9) years, with 20% being < 1 year of age. Mean weight was 20.7 (11.6) kg, and 63% of patients were female (Table 1). The most common indications for OPAT were pneumonia (*n* = 10, 27%), Pseudomonas eradication (*n* = 7, 17%), CVC infection (*n* = 5, 12%), and meningitis (*n* = 5, 12%) (Table 2). The mean length of hospital stay before the start of OPAT was 7.5 (4.6) days. Ninety percent of MCs were inserted in arm veins, most commonly the basilic vein (*n* = 26, 63%) (Table 2). The median number of days the MC was used for OPAT was 7 (5–10), and the median total MC dwell time was 10 (7.5–15) days (Table 2). The most frequently administered antibiotic was Ceftriaxone; 23 patients received Ceftriaxone either as single-drug therapy or in combination with another antibiotic. Other commonly used antibiotics were Tobramycine (*n* = 8) and Ceftazidime (*n* = 5).

**Table 1:** Patient characteristics in children with midline catheters used for OPAT (*n* = 41)

**Variable**	**All MCs** *n* = 41	**MC success** *n* = 31 (76%)	**MC failure** *n* = 10 (24%)	**ASD **^**a**^
Age 0–1 year, *n* (%)	8 (20)	5 (16)	3 (30)	
Age 1–5 years, *n* (%)	12 (29)	8 (26)	4 (40)	
Age > 5 years, *n* (%)	21 (51)	18 (58)	3 (30)	
Age, years, mean (SD)	5.9 (4.9)	6.4 (4.9)	4.1 (4.4)	0.49
Weight, kg, mean (SD)	20.7 (11.6)	22.3 (11.7)	15.9 (10.2)	0.58
Sex, female, *n* (%)	26 (63)	18 (58)	7 (70)	0.25
PICU admission, *n* (%)	5 (12)	4 (13)	1 (10)	0.09
In Hospital LOS, days, mean (SD)	7.5 (4.6)	7.9 (4.5)	6.3 (4.7)	0.35

*ASD* absolute standardized difference, *MC* midline catheter, *IQR* interquartile range, *OPAT* outpatient parenteral antibiotic therapy, *PICU* pediatric intensive care unit, *LOS* length of stay

^a^An ASD value > 0.71 is considered to indicate imbalance between the groups

**Table 2:** Characteristics of midline catheters (*n* = 41) used for pediatric OPAT

**Variable**	**All MCs** *n* = 41	**MC Success** *n* = 31 (76%)	**MC Failure** *n* = 10 (24%)	**ASD **^**a**^
MC insertion site:				1.36^b^
Basilic vein, *n* (%)	26 (63)	20 (65)	6 (60)	
Brachial vein, *n* (%)	4 (10)	3 (10)	1 (10)	
Cephalic vein, *n* (%)	6 (15)	6 (19)	0 (0)	
Saphenous vein, *n* (%)	4 (10)	1 (3)	3 (10)	
Median cubital vein, *n* (%)	1 (2)	1 (3)	0 (0)	
Left side, *n* (%)	17 (41)	15 (48)	2 (20)	0.62
Indication:				
- Pneumonia, *n* (%)	10 (24)	9 (29)	1 (10)	
- Pseudomonas eradication, *n* (%)	7 (17)	5 (16)	2 (20)	
- CVC infection, *n* (%)	5 (12)	5 (16)	0 (0)	
- Meningitis *n* (%)	5 (12)	1 (3)	4 (40)	
- Pyelonephritis, *n* (%)	4 (10)	2 (7)	2 (20)	
- Endocarditis, *n* (%)	2 (5)	1 (3)	1 (10)	
- Soft tissue infection, *n* (%)	2 (5)	2 (7)	0 (0)	
- Other, *n* (%)	6 (15)	6 (19)	0 (0)	
Catheter length, cm, mean (SD)	7.0 (1.9)	7.4 (1.8)	5.6 (1.6)	1.06
Catheter size, Fr, mean (SD)	2.7 (0.7)	2.8 (0.7)	2.3 (0.5)	0.82
Daily iv administrations, median (IQR)	1 (1–2)	1 (1–2)	1.5 (1–2)	0.11
MC days in OPAT, median (IQR)	7 (5–10)	7 (5–10)	4 (1.8–6.8)	1.05
MC days in hospital before OPAT	3 (1–6)	3 (2–6)	1.5 (0.8–3.3)	0.75
Total MC days, days median (IQR)	10 (7.5–15)	12 (8–16)	5 (4–11)	1.22
Electively removed, *n* (%)	27 (66)	27 (87)	0 (0)	3.66
MC failure per 1000 catheter days (CI)	28.5 (17.1–47.3)	9.5 (3.7–24.2)	143 (79.5–243.4)	

*ASD* absolute standardized difference, *CI* confidence interval, *MC* midline catheter, *Iv* intravenous, I*QR* interquartile range, *OPAT* outpatient parenteral antibiotic therapy

^a^An ASD value > 0.71 is considered to indicate imbalance between the groups

^b^When comparing upper body to lower body insertion site with groups of 4 and 37 children, respectively, an ASD value > 1.03 implies imbalance between groups

### Outcome of using MCs for OPAT

In 31 cases (76%, 95% CI 61–86%), the iv therapy could be successfully completed using only the MC. Four of these catheters were removed due to complications, but the physician in charge of the patient decided that no further iv therapy was needed. MC failure occurred in 10 cases (24%), as an additional venous catheter was needed to complete the intended therapy. This corresponds to an overall incidence rate of MC failure of 28.5 (95% CI 15.6–51.6) per 1000 catheter days (Table 2).

Patient age (6.4 vs 4.1 years) and weight (22.3 vs 15.9 kg) were similar in the MC success and the MC failure groups (Table 1). Catheter length (ASD 1.06) and size (ASD 0.82) both had ASD values > 0.71, indicating a possible increased risk of failure with shorter and thinner catheters (Table 2). MC failure occurred in 3 out of 4 catheters in the saphenous vein compared to 7 out of 37 catheters inserted in arm veins suggesting an imbalance in favor of arm vein insertion (Table 2). Dwell time for catheters in the MC success group was 12 days vs 5 days in the MC failure group, and the number of OPAT days was 7 vs 4 days (Table 2). Six children with MC failure needed hospital admission to receive a new venous catheter.

### Complications of MCs used for OPAT

Overall, 14 (34%, 95% CI 22–49%) MCs were subject to a complication, the most common being pain on injection (8 patients in the MC failure group, 2 patients in the MC success group) (Table 3). Pain on injection leads to the removal of the catheter in all cases. Leakage at the insertion site occurred in four (9.8%) patients, all in the MC failure group (Table 3). Overall, catheter occlusion occurred in two (both in the MC success group) and infiltration in four cases (two in each group). Only one case (2.4%) of symptomatic catheter-related venous thrombosis occurred (Table 3). No cases of MC-related bloodstream infection were found, and no catheters were accidentally extracted in the home environment (Table 3).

**Table 3:** Complications related to the use of midline catheters for pediatric OPAT (*n* = 41)

**Variable**	**All MCs** *n* = 41	**MC Success** *n* = 31 (76%)	**MC Failure** *n* = 10 (24%)
Pain on injection	10 (24)	2 (6.5)	8 (80)
Leakage at insertion site	4 (9.8)	0 (0)	4 (40)
Venous thrombosis	1 (2.4)	0 (0)	1 (10)
MC-related bloodstream infection	0 (0)	0 (0)	0 (0)
Catheter occlusion	2 (4.9)	2 (6.5)	0 (0)
Infiltration	4 (9.8)	2 (6.5)	2 (20)
Accidental extraction	0 (0)	0 (0)	0 (0)
MCs with any complication	14 (34)	4 (13)	10 (100)

*MC* midline catheter, *OPAT* outpatient parenteral antibiotic therapy. All data are presented as *n* (%)

## Discussion

In this retrospective study, we investigated the success rate of MCs for pediatric OPAT and described complications of the use of MCs in this setting. Our main finding is that OPAT could successfully be completed without the need for additional venous access in 76% of cases. Thirty-four percent of catheters were subject to a complication, with pain on injection in the MC being the most frequent.

A reliable vascular access is one of the key factors to delivering safe and efficient OPAT in pediatric patients. The use of MCs in the pediatric setting has increased in recent years. However, data on the relative benefits and drawbacks of MCs compared to other types of venous catheters is very limited [13, 14], and more knowledge of the expected dwell time and complication rate when using MCs for pediatric OPAT is necessary [15, 16]. For adult OPAT use, American guidelines issue a weak recommendation to use MCs for short courses of OPAT < 14 days [15]. However, neither American nor British guidelines could issue any recommendation regarding the use of MCs for pediatric OPAT due to a lack of scientific data [15, 16]. In our cohort, 76% of patients could complete their IV antibiotics without the need for additional venous access. This is similar to the success rate previously described by our group for pediatric MCs used in hospital [12]. Previous studies reporting data regarding completion of OPAT with a single catheter have mainly included PICC lines, and success rates in these reports vary between 82 and 95.5% [18–20]. In our study, 60% of patients in the MC failure group needed readmission to the hospital due to catheter failure, highlighting the role of reliable vascular access in the OPAT setting.

MC failure occurred in 3 out of 4 catheters in the saphenous vein compared to 7 out of 37 catheters inserted in arm veins, suggesting an imbalance in favor of arm vein insertion. Imbalances also suggested unfavorable outcomes of shorter and smaller-sized catheters. Saphenous vein insertion as well as shorter catheters have been shown to increase the risk of MC-related venous thrombosis [12], possibly contributing to an increased risk of failure. Moreover, shorter catheters are likely to have a smaller part of the catheter inside the vein possibly increasing the risk of catheter migration outside the vein. Another possible advantage of the use of longer catheters is a tip position in a more proximal vein with greater blood flow and hemodilution.

Data regarding the rate of complications to pediatric MCs in this setting is very limited. We found that 14/41 MCs (34%) were subject to a complication. Two studies presenting data on small numbers of MCs used for OPAT reported complications in 3/7 (43%) [21] and 3/14 MCs (21.4%) [22], respectively. In the hospital setting, 51% of pediatric MCs had a complication [12]. However, approximately one-third of these complications were asymptomatic catheter-related venous thrombosis that were only detected on screening ultrasound. Disregarding asymptomatic events, which could not be detected in this retrospective report, the rate of complications was similar between the two studies. No catheter-related bloodstream infections were found. This is in line with earlier data indicating that catheter-related bloodstream infections are uncommon with pediatric MCs.

Previous data on the rate of vascular access-related complications during OPAT mainly includes PICC lines and have reported complication rates varying from 8 to 33.3% [18, 19, 21, 23]. However, there is a substantial heterogeneity between different OPAT services concerning case mix, age of patients, and routines for vascular access management. This fact makes it hard to draw firm conclusions when comparing the failure rate and the rate of complications between reports from different institutions. Data from adult studies indicate a higher rate of complications with MCs compared to PICC lines in the OPAT setting [24, 25]. There is a need for randomized controlled trials systematically investigating the benefits and drawbacks of different types of vascular access when used in the pediatric OPAT setting.

The overall incidence rate of MC failure (28.5 per 1000 catheter days) was somewhat lower, and median MC dwell time (10 days) was longer than previous reports on pediatric MCs used in the hospital setting [12]. However, it should be kept in mind that these variables are strongly influenced by the indication for treatment and the intended duration of therapy.

To our knowledge, this is the first study describing the use of MCs for pediatric OPAT. Our comprehensive electronic records allowed for a thorough evaluation including consecutive patients with few exclusion criteria. However, this study also has some limitations that need to be considered. The retrospective design only allowed us to identify symptomatic complications. Midline catheters were not compared to other catheter types in a randomized manner. This was a single-center study from a tertiary pediatric center, and our results may not be applicable to other centers, age groups, or catheter types. Moreover, a larger sample size would naturally have given a more precise estimation of MC success rate and complications.

## Conclusions

In this retrospective study, the use of MCs led to the successful completion of therapy in 76% of cases. The performance of MCs in the OPAT setting was similar to previous data from our hospital setting. Factors associated with MC failure were saphenous vein insertion as well as shorter and smaller gauge catheters. Midline catheters could be an alternative to peripherally inserted central venous access for pediatric OPAT, but further randomized controlled studies are necessary to investigate the relative advantages and disadvantages of different vascular catheter types in this setting.

## Data Availability

The data used in the study are available from the corresponding author on reasonable request.

## Abbreviations

CI: Confidence interval
CVC: Central venous catheter
IQR: Interquartile range
MC: Midline catheter
OPAT: Outpatient parenteral antimicrobial therapy
PIVC: Peripheral intravenous catheter
SD: Standard deviation
